# New Approach of QuEChERS and GC-MS Triple-Quadrupole for the Determination of Ethyl Carbamate Content in Brazilian *cachaças*

**DOI:** 10.3389/fnut.2018.00021

**Published:** 2018-04-06

**Authors:** Tatiane Melina Guerreiro, Kumi Shiota Ozawa, Estela de Oliveira Lima, Carlos Fernando Odir Rodrigues Melo, Diogo Noin de Oliveira, Simone Pereira do Nascimento Triano, Rodrigo Ramos Catharino

**Affiliations:** ^1^Innovare Biomarkers Laboratory, School of Pharmaceutical Sciences, University of Campinas, Campinas, Brazil; ^2^Agilent Technologies (Brazil), São Paulo, Brazil

**Keywords:** alcoholic beverages, *cachaça*, ethyl carbamate, gas chromatography–mass spectrometry, QuEChERS

## Abstract

*Cachaça* is a popular spirit produced in Brazil, obtained by distillation of fermented sugar cane. Among the contaminants arising from production, ethyl carbamate is a carcinogenic compound that occurs naturally in fermented foods and beverages; in Brazil, the maximum limit established by current legislation is 150 µg L^−1^. Quality control is usually performed using gas chromatography; however, robustness and reproducibility of quantitative results may be severely impaired, as the addition of 6–30 g L^−1^ of sucrose is a common procedure for taste standardization, directly interfering in the results. This work describes the development of a novel method to improve ethyl carbamate quantification in *cachaças* using a new approach of QuEChERS extraction based on salting-out phenomenon, to effectively separate ethanol from sugar-containing water. Eighteen different brands of *cachaça* were analyzed. The proposed methodology was able to eliminate components that contaminate the sample flow path in the gas chromatography system, while improving precision and accuracy by using a triple-quadrupole approach, in comparison with the methodology usually employed: direct analysis of *cachaça* samples with no sample prep. Results indicate that this approach is more effective due to the removal of sugar content, with no impact in costs per analysis.

## Introduction

*Cachaça* is the typical and exclusive denomination of a sugar cane spirit produced in Brazil. Characteristics that define this product are the alcohol content reaching 38–48% ABV (alcohol by volume) at 20°C and obtainment by distillation of fermented sugar cane juice (1). The manufacturing process of *cachaça* is not standardized in several aspects. The first is regarding the type of sugar cane used, since there are several varieties currently used, which present great similarity in their composition (2). In addition, grains such as corn or rice may also be added to the must in order to provide nutrients for better development of yeasts responsible for fermentation (3). Finally, the equipment used may be different as well, since the distillation may be carried out in columns of distillation made from stainless steel, in the case of industrial production, or in copper stills, in the case of artisanal production (4). The aging process in barrels of wood is similar to that applied in the manufacturing of wine, where oak, balm, *jequitibá, jatobá*, or *amburana* barrels may be used. This last process is optional and aims to provide aromas, flavors, and colors from the wood to the *cachaça* (5). Regarding the sensorial characteristics, it is observed that high-quality *cachaça* has a characteristic aromatic bouquet from sugar cane, as well as aromas and unique flavors. However, in addition to the organoleptic aspects, *cachaça* must meet some chemical parameters to assure that, with moderate consumption, the product causes no health hazards. Contaminants such as methanol, sec-butanol, *n*-butanol, copper, and ethyl carbamate, as well as acetic acid, an off-flavor responsible for the acidity of *cachaça*, are key species that are directly linked to quality impairment of this distillate (6, 7).

According to a 2007 assessment by IARC (International Agency for Research on Cancer), ethyl carbamate was considered carcinogenic in laboratory animals, and therefore, has been listed as a carcinogen for humans, belonging to Group 2A on the Classification of Carcinogenic Substances (8, 9). This compound is an ethyl ester of carbamic acid that is formed naturally in fermentation processes, which may be derived from several different precursors, such as hydrocyanic acid, urea, citrulline, and *N*-carbamyl by reaction with ethanol (4, 9–12). Due to this feature, it is easily found in various types of food and beverages, such as spirits, wine, beer, bread, shoyu sauce, and yogurt (13, 14). The consumption of ethyl carbamate only from food sources may be deemed insignificant. However, by adding the intake of alcoholic beverages on a regular basis, exposure to this compound increases; for example, *cachaça* figures as the third most consumed distilled beverage in the world, and in Brazil it is the second most consumed alcoholic beverage, after beer (15). Given its wide consumption, measures must be taken regarding the reduction of ethyl carbamate concentration in alcoholic beverages. Canada, through the “Health and Welfare Department” in 1985, was the first country to determine concentration limits of ethyl carbamate in wines, distilled beverages, fruit distillates, and liqueurs. Therefore, this became a reference for the United States and European Union, regarding the legislation on this subject (16). In 1990, the FDA published a note limiting ethyl carbamate content in American whiskeys in 125 µg L^−1^ (17). According to the Brazilian Ministry of Agriculture, Livestock, and Supply, the limit for this contaminant in *cachaças* is 150 µg L^−1^ (1). The importance of controlling ethyl carbamate levels, therefore, must be understood as an issue concerning not only health aspects, but also economic ones, as any out-of-specification result may represent a barrier to exporting Brazilian *cachaça* to both American and European markets.

Many different approaches using mass spectrometry (MS) have been developed in the recent years, assessing a great diversity of quality parameters from *cachaça* (18–20), as well as many protocols for ethyl carbamate analysis and extraction (9). The use of gas chromatography (GC) coupled with single-quadrupole MS is a technique widely used for the analysis of this contaminant, as it is capable of accurately promoting both identification and quantification in food and beverage samples (9). However, there is a common procedure of sucrose addition in sugar cane spirits to reduce the alcoholic flavor of the beverage and standardize its taste, improving its final sensory quality. In the case of *cachaça*, the Brazilian legislation allows the addition of up to 6 g L^−1^, as procedure of standardization. Nonetheless, if the label states “sweetened,” the product may contain up to 30 g L^−1^ of sugar (21). As GC-MS is the method of choice for the quality control of *cachaça*, high sucrose content may easily contaminate the GC flow path, thus making the maintenance of the equipment more frequent, and compromising the accuracy of results. In this sense, the development of a methodology capable of coping with exceeding amounts of sucrose present in *cachaça*, while maintaining the precision and accuracy of the analysis, became necessary.

Related to the preparation of samples, carbamates usually occur at very low concentrations and in complex matrices, as the *cachaça* samples, because of that they cannot be determined without some preliminary sample pretreatments. A good procedure of sample preparation should provide the preconcentrating of analytes and also the clean-up, improved in this way the determination of the analyte of interest. Recently, the search for miniaturized methods that are efficient and do not use large amounts of toxic solvents to perform the extraction has been in focus. Thus, different microextraction procedures have been developed as alternatives like the QuEChERS (quick, easy, cheap, effective, rugged, and safe) methodology, which has been widely used in the pretreatment of complex samples. This technique is based on a two-step method where it is first performed an extraction step based on partitioning *via* the salting-out phenomenon, which is responsible for the equilibrium between an aqueous and an organic layer. The second step is a solid phase dispersive extraction, which involves the cleanup of the sample using combinations of different kinds of sorbents salts or primary-secondary amines with the objective to remove interfering substances (22, 23).

In this work, we present a new approach of QuEChERS, based only on the salting-out extraction, as a way to eliminate residues of sucrose and water for samples of several brands of Brazilian *cachaça*, which was further corroborated by an experiment using liquid chromatography (LC), without compromising the identification and quantification of ethyl carbamate. Additionally, we compared this new methodology developed by our group with the current recommended procedure for the analysis of ethyl carbamate.

## Materials and Methods

### Reagents and Standards

The standard of ethyl carbamate (99.0%) was purchased from Sigma Aldrich (MO, USA). Potassium carbonate (99.6%) was purchased from Neon (Suzano, Brazil). C*achaças* of different Brazilian brands (9 commercial) and common sugar (sucrose) were purchased from commercial establishments in the city of Campinas, Brazil. The craft *cachaças* (9) were purchased from different artisanal producers in the countryside of the States of São Paulo and Minas Gerais, Brazil. Ethanol was purchased from Panreac AppliChem (Barcelona, Spain), Acetonitrile was purchased from J.T. Baker (Xalostoc, Mexico); both were LC grade and were used with no further purification. Deionized water was obtained with a Milli-Q system (Millipore, USA).

### Sample Preparation—New Approach of QuEChERS

In order to analyze samples of *cachaça*, a new methodology of QuEChERS was developed. We performed a salting-out using 5 g of potassium carbonate added to 10 mL of sample and vortexed for 20 s. The tube was left to rest for 2 minutes until phase separation and, after that, 1 mL of the alcoholic phase was transferred to a vial and injected into Agilent GC/MS 7890/7000 Triple Quadrupole system. For the analysis performed into an Agilent LC/MS/MS 6460 Triple Quadrupole system these same samples were diluted 10 times (in water). All samples were prepared in triplicates.

### LC-MS Analysis

In order to check the reduction of sucrose amount, samples were analyzed before and after the QuEChERS with potassium carbonate. An aliquot of 100 µL of samples, with or without QuEChERS proceeding, was diluted with 900 µL of deionized water. A volume of 0.5 µL was injected at 65°C into an Agilent LC/MS/MS 6460 Triple Quadrupole system. The two mobile phases used were (A) with 90% of water, and (B) with 10% of acetonitrile. The flow rate was 0.350 mL min^−1^ and the column used was an Agilent Hi-Plex Ca (USP L19) 8 µm, 4.0 mm × 250 mm. The mass spectrometer was set to electrospray ionization mode (ESI) with capillary voltage at 5 kV. All samples were prepared in triplicates and each of them were analyzed in triplicate.

### GC-MS Analysis

A stock solution of 2,000 mg L^−1^ was prepared using ethyl carbamate standard diluted in ethanol. To build the calibration curve, an aliquot of the stock solution was diluted in ethanol to yield a calibration solution with concentration of 10 mg L^−1^ of ethyl carbamate. From this calibration solution, new dilutions were performed in order to obtain six concentration points chosen for the construction of the external calibration curve: 50, 100, 200, 500, 1,000, and 1,500 µg L^−1^. Concerning matrix interference, an experiment with internal standard addition was performed, using a sandwich injection. This experiment was performed in order to verify whether the QuEChERS approach would clean up the sample by decreasing matrix interference and not impairing the ethyl carbamate determination in the analyzed samples.

The instrument used was an Agilent GC/MS 7890/7000 Triple Quadrupole system with Electron Ionization mode (EI). A volume of 2 µL of sample was injected in split mode 1:5 at 280°C as inlet temperature. Nitrogen was used as collision gas with flow rate of 1.5 mL min^−1^; Helium was used as carrier gas at flow rate of 1 mL min^−1^ and as quench gas at a flow rate of 2.25 mL min^−1^. The oven started at 90°C for 1 min and the temperature increased at a rate of 10°C min^−1^ until the final temperature of 185°C, with a total running time of 10.5 min. The column used was Agilent VF-WAXms 0.25 µm, 30 m × 0.25 mm and the mass spectrometer source temperature was set to 300°C, while quadrupole Q1 and Q2 at 180°C. The Multiple Reaction Monitoring transitions used were for the quantifier ion 62 → 44 with collision energy of 15 eV and qualifier ion 74 → 44 with collision energy of 5 eV. All samples were analyzed in triplicates.

### Sandwich Injection Applied to GC-MS Analyses

**Figure 1:**
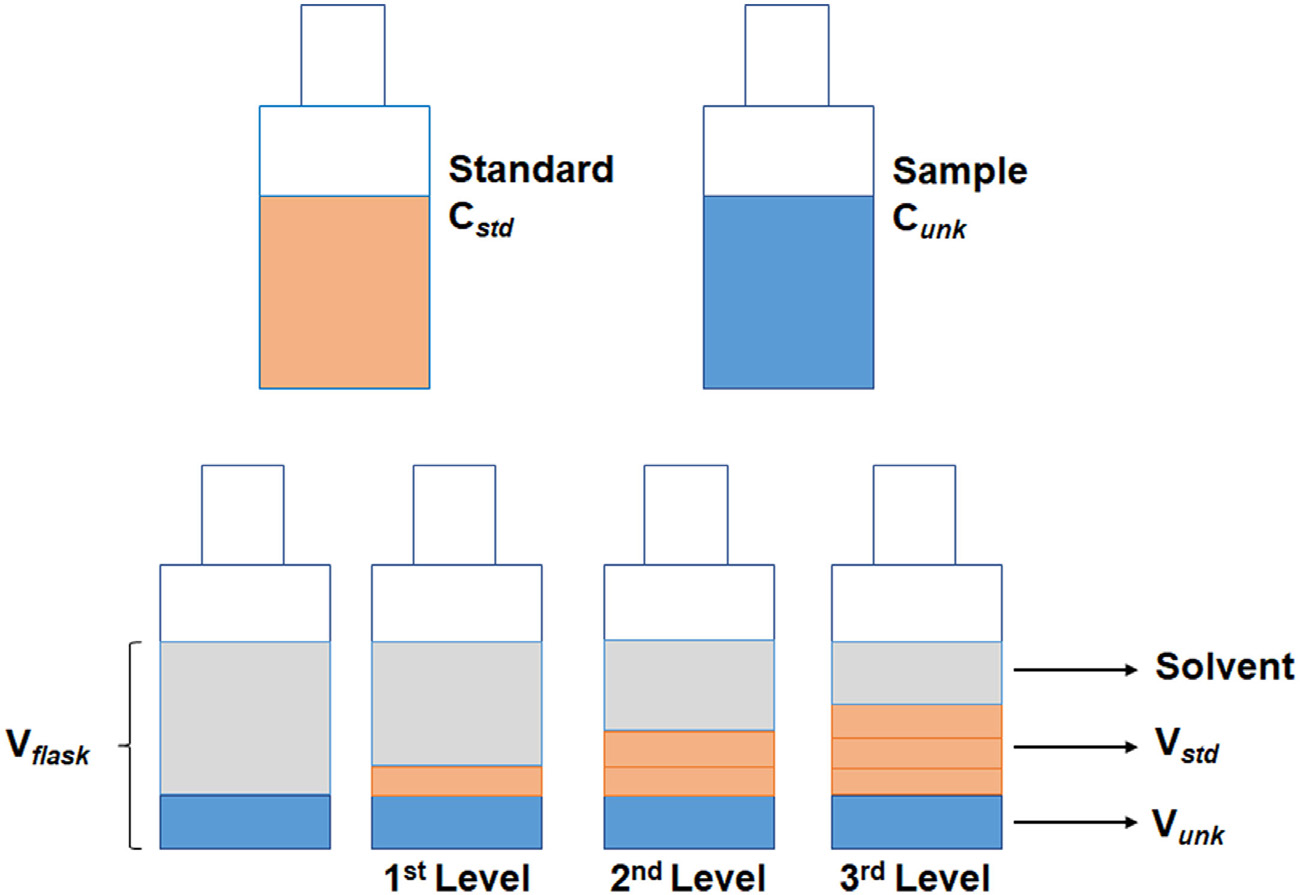
Scheme of the solution preparation for the standard addition method.

**Figure 2:**
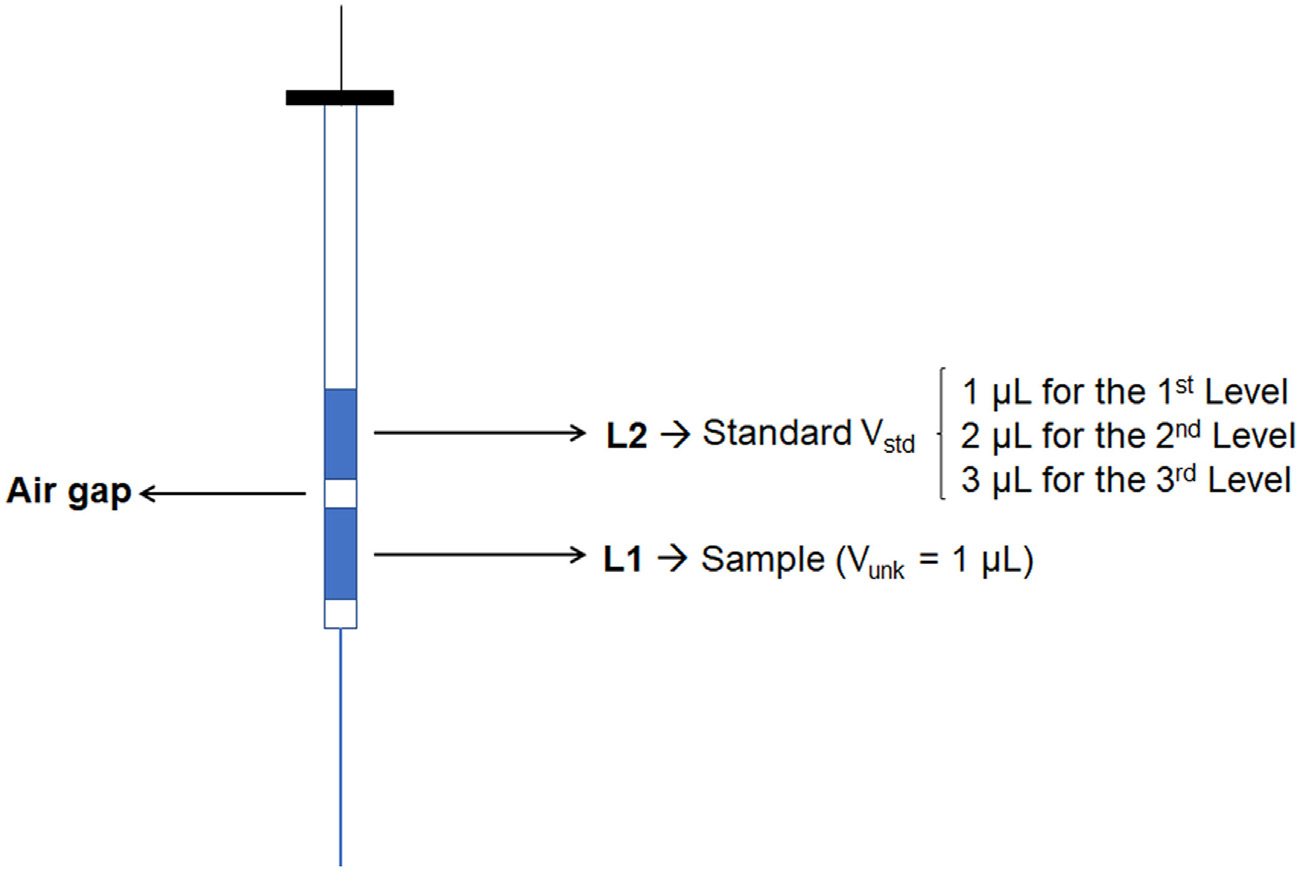
Sandwich injection scheme.

The injection of the sample using the standard addition method was performed by creating a different acquisition method for each calibration level. The first level was composed only by the sample volume to be analyzed, thus the injection was 1 µL of sample in split mode (1:5). The second level was the mixture between the sample and one aliquot of standard; hence, the injection was performed by programming the autosampler to take 1 µL of sample and 1 µL of standard. The standard vial was prepared at concentration of 100 µg mL^−1^. The third level consisted of taking 1 µL of sample and 2 µL of standard. Finally, the fourth level took again 1 µL of sample and 3 µL of standard (Figures 1 and 2). The standard was positioned in such a position in the autosampler that the auto-injector could take the aliquot of standard solution always at the same place. Upon each injection, the syringe was washed four times with ethanol (40%) first, and six times with a deionized water: acetonitrile solution (80:20) after the injection, both at the maximum volume of 10 µL.

## Results and Discussion

### Part I: Sucrose Content Verification by LC/MS/MS

**Figure 3:**
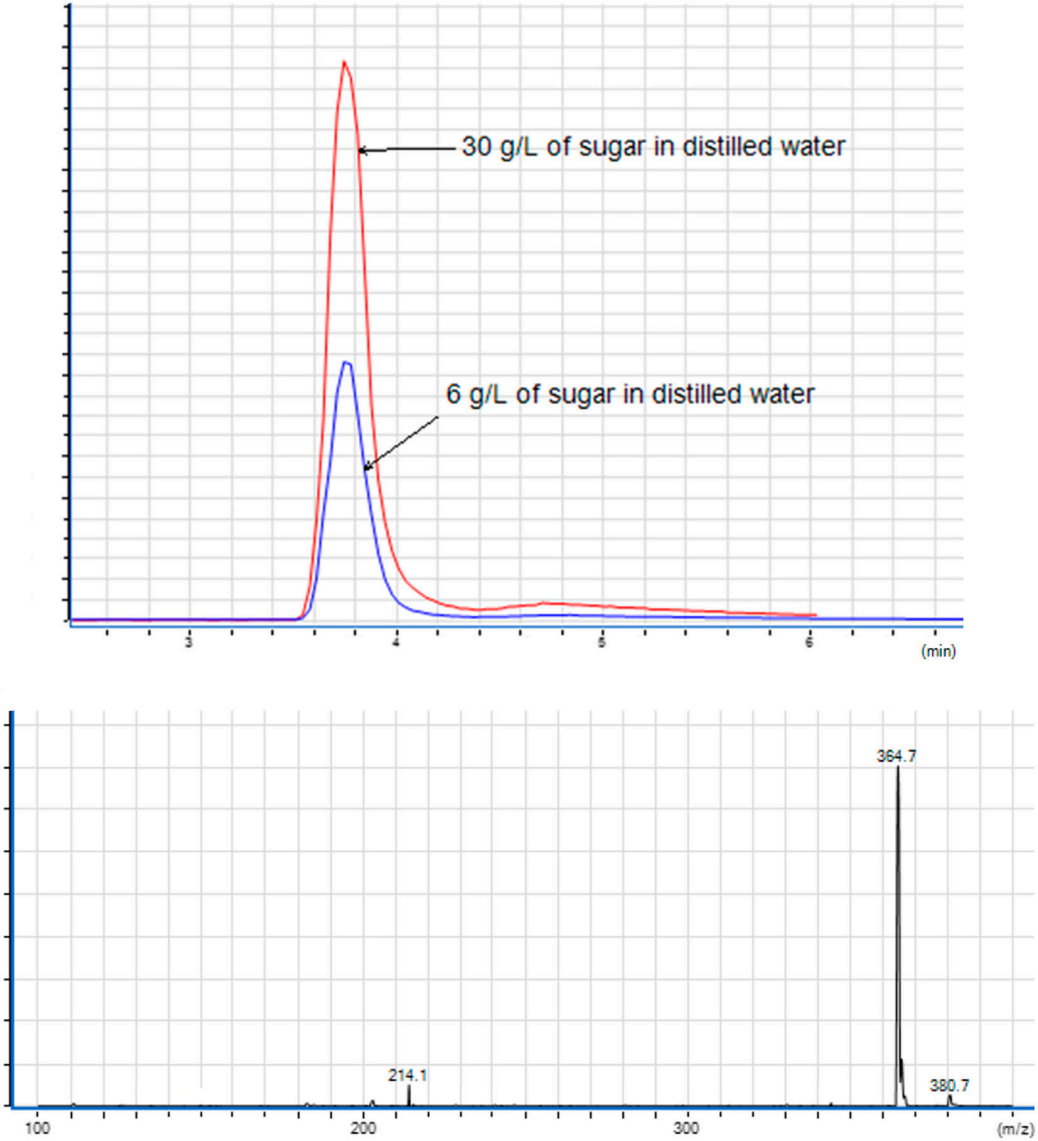
Extracted ion chromatogram of solutions with 6 and 30 g L^−1^ of sugar and mass spectrometry spectrum of 365 (sucrose).

**Figure 4:**
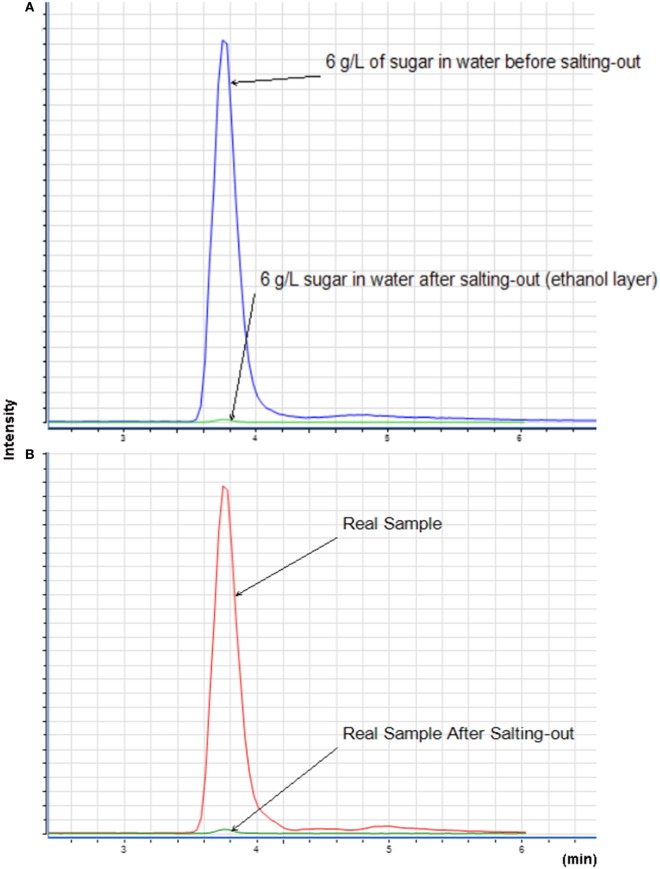
Extracted ion chromatogram of 6 g L^−1^ of sugar **(A)** and with a sample of *cachaça* **(B)** before and after QuEChERS extraction, injecting only the organic layer for both samples.

The sugar present in *cachaça*, sucrose, is a naturally occurring saccharide found in sugar cane. The solubility of sucrose in water is 1 g for 0.5 mL, and in ethanol is 1 g for 170 mL. With the purpose of simulating the amount of sucrose in *cachaça* samples, mock solutions of 0.3 and 0.06 g of sucrose in 10 mL of water were prepared, emulating the maximum concentrations for not labeled (6 g L^−1^) and labeled (30 g L^−1^) sugar addition. Figure 3 shows the Extracted Ion Chromatogram (EIC) 365 of sucrose (24) of the 6 and 30 g L^−1^ preparations. Integration of the EIC 365 peak was performed and compared before and after the QuEChERS extraction. Elimination of most of the sugar was evident, since the solubility of sucrose in water is higher than in ethanol, as shown in Figure 4 and in the data available in Table 1. This happened because when a salt is added to an aqueous solution of a soluble organic solvent, the intermolecular interactions are affected by the ionization of the salt because of the stronger affinity between the ions and the water molecules. This reflects in decreased availability of water molecules for the third component (i.e., organic solvent). Therefore, the organic solvent is forced to increase its intermolecular interactions, and subsequently, at threshold concentrations of the ionized species, the organic solvent is excluded from the rest of the solution as a separate phase (25). This phenomenon is known as salting-out and was applied in the development of this new approach of QuEChERS extraction described here. In the present case, separation of ethanol from water by the QuEChERS approach eliminates most of the sucrose due to its solubility, which was verified using an LC/MS triple quadrupole system.

**Table 1:** Sugar percentage removal by extracted ion chromatogram peak area.

	Area before salting-out	Area after salting-out	Removal percentage
Control 6 g L^−1^	83,180,455.52	405,192.79	99.51
Control 30 g L^−1^	193,679,686.4	1,906,989.36	99.01
*Cachaça* Sample	82,252,299.47	706,338.66	99.10

### Part II: Matrix Interference in GC/MS/MS and Standard Addition

Matrix interference is another issue involved in the analysis of ethyl carbamate in alcoholic beverages using GC-MS. Even after sample cleanup by a diatomaceous earth column, the identification of ethyl carbamate often cannot be ascertained with confidence by the selected ion monitoring (SIM) mode, due to inconsistent ratios of the SIM ions 62, 74, and 44 *m/z*, since the qualifier ions are highly susceptible to matrix interference (26). Therefore, the use of triple-quadrupoles for improved sensitivity and specificity may clarify this interference.

**Figure 5:**
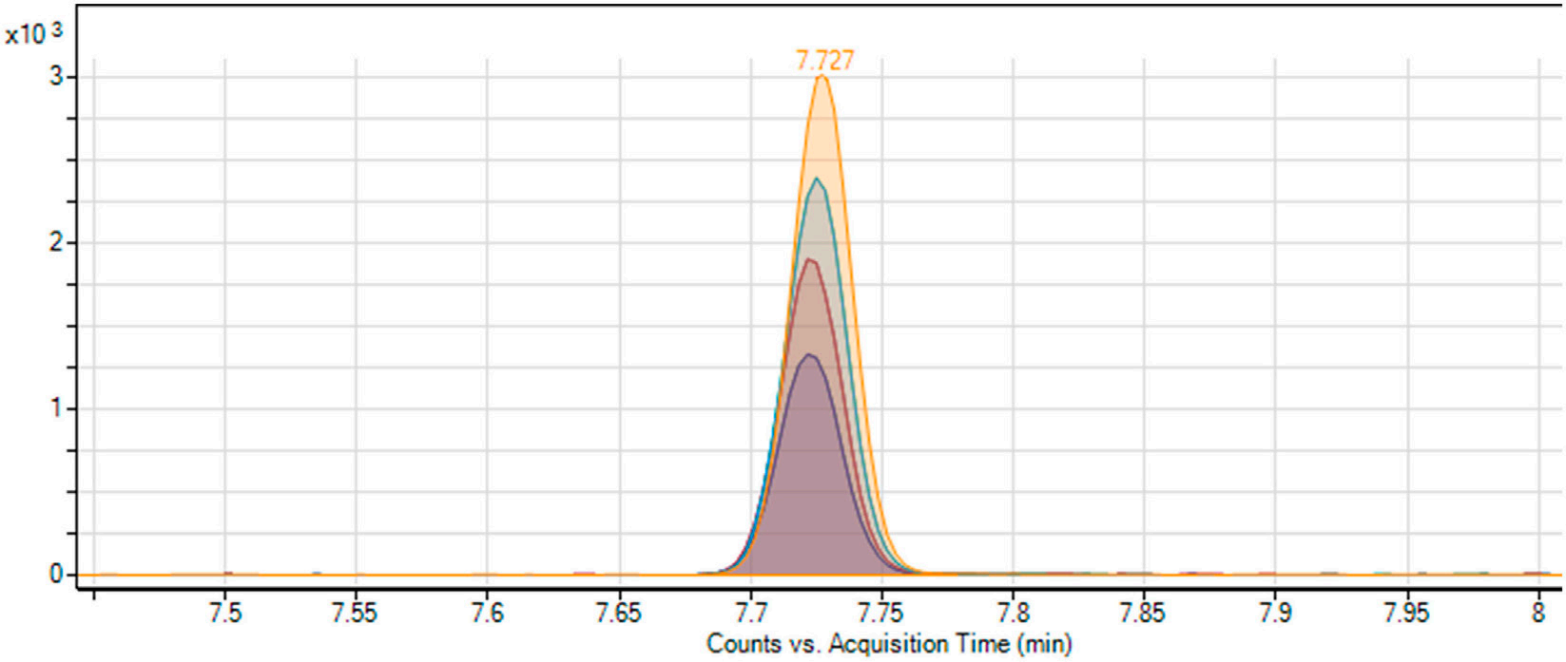
Overlapping chromatogram of standard addition method, which shows the gradual increase of the peak area of ethyl carbamate.

An alternative with the use of mixtures of ethanol and water might be an option for blank samples, but they cannot comprehend all the potential matrix interference to the analysis. Thus, the standard addition method is suggested to perform a quantitative analysis approach where the standard is added directly to the aliquots of the analyzed sample. This approach may be applied in cases where a suitable blank sample is not available for calibration, as with *cachaça* samples. Moreover, the method of standard addition can assist in identifying a target analyte in any given chromatogram, as only the target peak increases after the addition. Figure 5 shows the overlapping chromatogram of samples added and not added with ethyl carbamate standard. The only clear disadvantage of this method is the laborious task to prepare the solution to determine the calibration curve. To perform standard addition, it is required to prepare a set of solutions that contains a same volume of sample and increasing volume of standard solution, as shown in Figure 1. The methodology is based in the addition of a constant volume (V*unk*) of sample to each of the volumetric flasks, with volume (V*flask*), then adding a series of increasing volumes of stock solution (V*std*). Finally, each flask is made up to the mark with solvent and mixed.

This process was further simplified by the use of sandwich injection (Figure 2) performed by automatic injector of the GC system. This injection consists of taking an aliquot of sample volume and standard solution volume at the same time injecting both the solution together, thus avoiding the preparation of the standard solution with varied concentrations. Since the solvent is evaporated in GC injection port, the calibration curve is based on mass injected in the column, instead of the concentration established in the volumetric flask. Results compare the final concentration (FC) obtained by standard addition and external calibration method.

### Part III: Quantification of Ethyl Carbamate

The blank samples were prepared with ultrapure water and ethanol, with a FC of 40% ethanol. The samples were spiked with concentrations of 50, 100, and 200 µg L^−1^ of the standard. As the solubility of ethyl carbamate in ethanol (1.2 g mL^−1^) is higher than in water (0.1 g mL^−1^), the FC in ethanol, after the QuEChERS extraction step, will be proportional to that volume. For example, when adding 100 µL of a solution with a concentration of 10 ng µL^−1^ of ethyl carbamate, it is in the same proportion as 1,000 ng of ethyl carbamate in 10 mL of sample solution. After the QuEChERS extraction step, 1,000 ng of ethyl carbamate will be present almost exclusively in the ethanol layer. Thus, the FC in 1 mL of the upper layer (ethanol) will be approximately of 250 ng µL^−1^, as shown in equation below. This was corroborated using the calibration curve prepared in blank matrix:

$$C=1,000\text{ ng}/4\text{ mL}\left(\text{ethanol}\right)=250\text{ ng }µ{\text{L}}^{-1}\text{ or }250\;µ{\text{g L}}^{-1}.$$

The recovery (Rec) was verified by spiking 50, 100, and 200 µL of intermediate solution in 10 mL blank samples already corrected as per the equation above. For this reason, the expected concentration for 50, 100, and 200 µg L^−1^ would be 125, 250, and 500 µg L^−1^, respectively.

Although SD is higher by using the standard addition method, recuperation results are close to 100% when this method is applied to real sample, according to Table 2. This is because all matrix constituents were considered, including any inherent interference, in quantitation results. For real *cachaça* samples, water composition may vary from 38 to 48%. Considering that, Rec was within the acceptable range of 70–120% according to document SANTE (27).

**Table 2:** Recovery (Rec) results by standard addition and external calibration methods (*n* = 3).

	Standard addition			External calibration		
	Day 1 (%)	Day 2 (%)	Σ (%)	Day 1 (%)	Day 2 (%)	Σ (%)
Rec (50 ppb)	124	116	120	98	91	94
RSD	3	9	7	0	2	4
Rec (100 ppb)	104	106	105	81	84	83
RSD	8	10	9	3	1	3
Rec (210 ppb)	85	103	94	79	81	80
RSD	1	1	9	2	0	2

**Figure 6:**
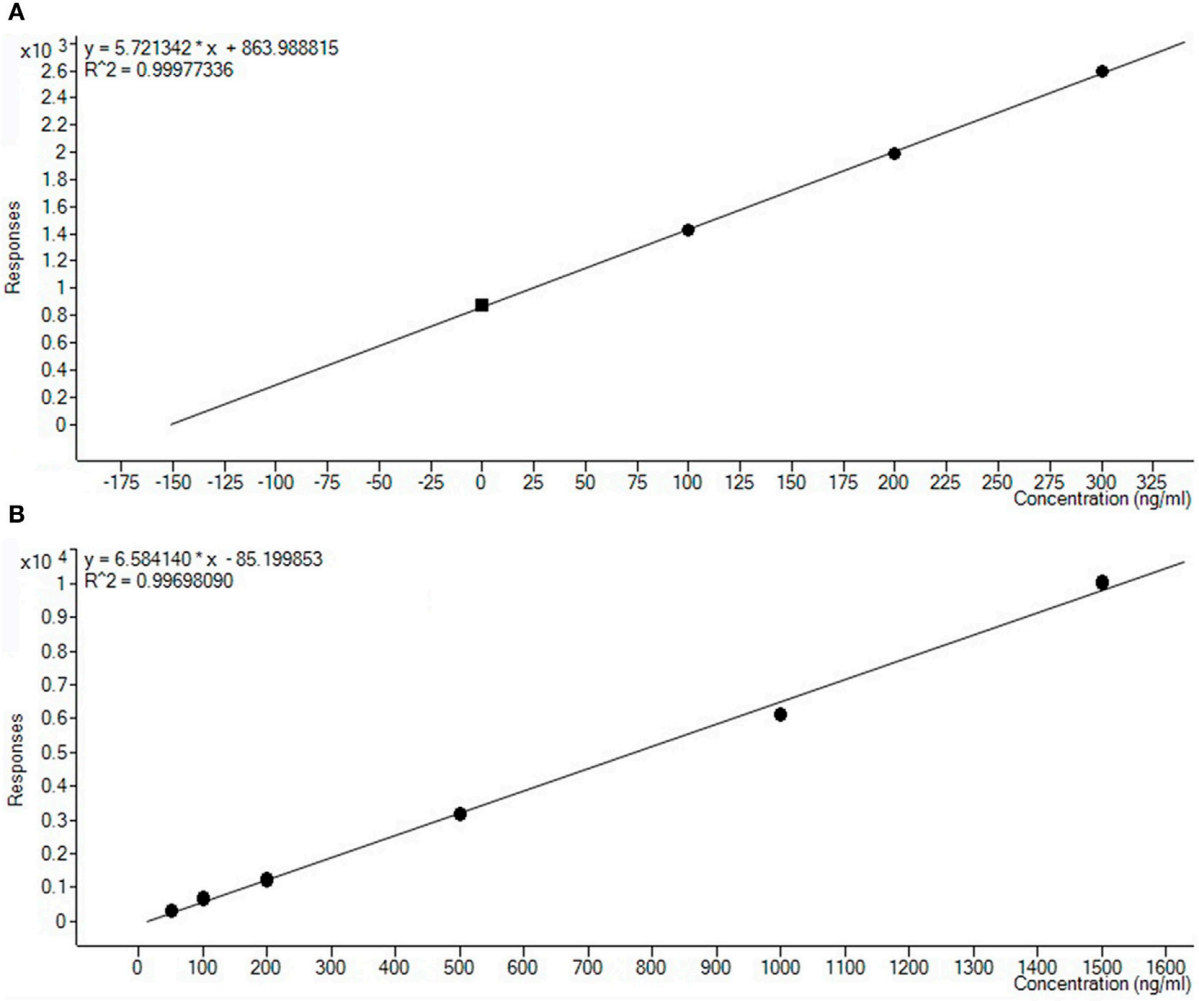
Comparison between the standard addition curve **(A)** and the external calibration curve **(B)** for quantification of the ethyl carbamate.

For quantification of the ethyl carbamate in the actual samples of *cachaça*, two calibration curves, the external calibration curve (related to the current recommended procedure) and the standard addition curve (new methodology), were used to make it possible to compare and validate the proposed methodology. The retention time of the analyte was 7.727 min, and remained stable across all different samples, with a variation coefficient equal 0.02% (Figure 5). A calibration curve with the standard addition was prepared by the linear regression method. The amount of the analyte in the sample without addition of ethyl carbamate was calculated from the calibration curve. A correlation coefficient of at least 0.995 generally indicates acceptable characterization of the curve; the proposed method was capable to obtain excellent linearity, with typical values for the correlation coefficient (*R*^2^) around 0.999 (Figure 6).

As shown in Table 3, out of the 18 samples tested, 9 commercial and 9 craft, only one showed an amount of ethyl carbamate above that allowed by the Brazilian legislation. In addition, we may observe that there is not a significant error between the measurements among commercials brands. Regarding the craft products, higher values of error were observed between the measurements presented, which may be related to the method of manufacturing this type of *cachaça*; since this is an artisanal product, it may be expected that there is less homogeneity among the final products. In this way, it is possible to verify that the developed methodology could be applied instead of the usual methodology because, despite the inclusion of sample preparation, it is simple and fast due to the use of QuEChERS.

**Table 3:** Mean concentration (MC, *n* = 3), absolute error of the measurements (*n* = 3), and final concentration (FC) of ethyl carbamate in the tested *cachaça* samples [commercial (Comm) and craft].

Sample	MC	Error (%)	FC (ppb)
Comm 1	212.4429	0	53.1107
Comm 2	117.1811	0	29.2953
Comm 3	26.8651	2	6.7163
Comm 4	245.4955	2	61.3739
Comm 5	250.6639	1	62.6660
Comm 6	186.6799	2	46.6700
Comm 7	105.0681	0	26.2670
Comm 8	493.4672	6	123.3668
Comm 9	250.0942	0	62.5236
Craft 1	530.7167	3	132.6792
Craft 2	604.5791	5	151.1448
Craft 3	306.7847	1	76.6962
Craft 4	467.9504	29	116.9876
Craft 5	176.5997	5	44.1499
Craft 6	61.9448	1	15.4862
Craft 7	205.4501	17	51.3625
Craft 8	54.0808	2	13.5202
Craft 9	138.3081	1	34.5770

## Conclusion

The issue of ethyl carbamate concentration in beverage led to a required monitoring during manufacturing process and in the final product. Beverage industries must analyze each production batch to check if it is in accordance with the maximum allowed limit. In order to control all the production, the method must be easy and robust as possible. The presence of sucrose in many *cachaças* makes this task very complicated, in addition to other matrix components that may interfere in the analysis. This study presented a simple and effective methodology for identification and quantification of ethyl carbamate in *cachaças*, presenting a new approach of QuEChERS based on the salting-out phenomenon, with leads to minimal sample preparation, and setup of standard addition method that may be easily prepared by an automatic injector system. This new methodology showed an increased level of efficacy, with a cost per analysis of approximately US$ 1.00, which can be considered a cost-effective approach when compared to the current recommended methodology. In this way, the demonstrated method can be understood as a valid alternative for the identification and quantification of ethyl carbamate in *cachaças*.

## Author Contributions

TG, KO, and ST performed experiments. TG and KO wrote the manuscript and performed data analysis. CM, DO, and EL performed manuscript review. RC idealized all experiments and managed the research group.

## Conflict of Interest Statement

The authors KO and ST were employed by company Agilent Technologies. All other authors declare no competing interests of any kind.
